# Novel Triazole-Quinoline Derivatives as Selective Dual Binding Site Acetylcholinesterase Inhibitors

**DOI:** 10.3390/molecules21020193

**Published:** 2016-02-05

**Authors:** Susimaire P. Mantoani, Talita P. C. Chierrito, Adriana F. L. Vilela, Carmen L. Cardoso, Ana Martínez, Ivone Carvalho

**Affiliations:** 1 School of Pharmaceutical Sciences of Ribeirão Preto, University of São Paulo, Ribeirão Preto 14040-903, Brazil; smp@usp.br (S.P.M.); tali@fcfrp.usp.br (T.P.C.C.); 2 Departamento de Química, Grupo de Cromatografia de Bioafinidade e Produtos Naturais, Faculdade de Filosofia Ciências e Letras de Ribeirão Preto (FFCLRP), Universidade de São Paulo, Ribeirão Preto 14040-901, Brazil; avilela@usp.br (A.F.L.V.); ccardoso@ffclrp.usp.br (C.L.C.); 3 Centro de Investigaciones Biológicas (CIB-CSIC), Madrid 28040, Spain

**Keywords:** Alzheimer’s disease, acetylcholinesterase, selective dual binding site inhibitors, click chemistry, triazole-quinoline derivatives

## Abstract

Alzheimer’s disease (AD) is the most prevalent neurodegenerative disorder worldwide. Currently, the only strategy for palliative treatment of AD is to inhibit acetylcholinesterase (AChE) in order to increase the concentration of acetylcholine in the synaptic cleft. Evidence indicates that AChE also interacts with the β-amyloid (Aβ) protein, acting as a chaperone and increasing the number and neurotoxicity of Aβ fibrils. It is known that AChE has two binding sites: the peripheral site, responsible for the interactions with Aβ, and the catalytic site, related with acetylcholine hydrolysis. In this work, we reported the synthesis and biological evaluation of a library of new tacrine-donepezil hybrids, as a potential dual binding site AChE inhibitor, containing a triazole-quinoline system. The synthesis of hybrids was performed in four steps using the click chemistry strategy. These compounds were evaluated as *h*AChE and *h*BChE inhibitors, and some derivatives showed IC_50_ values in the micro-molar range and were remarkably selective towards *h*AChE. Kinetic assays and molecular modeling studies confirm that these compounds block both catalytic and peripheral AChE sites. These results are quite interesting since the triazole-quinoline system is a new structural scaffold for AChE inhibitors. Furthermore, the synthetic approach is very efficient for the preparation of target compounds, allowing a further fruitful new chemical library optimization.

## 1. Introduction

Alzheimer’s disease (AD) is the most common form of dementia regarded as comprising memory loss, cognitive impairment, and difficulty in thinking and problem-solving. These symptoms worsen over time, becoming severe enough to interfere with daily common activities, thus necessitating the help of caregivers. Although the etiology of AD is not completely known, some evidence of extracellular β-amyloid (Aβ) deposits (senile plaques) and τ-protein aggregation (hyperphosphorylation of tau protein) are found in the brain of AD patients, in addition to the selective loss of cholinergic neurons, resulting in a deficit of acetylcholine (ACh) in areas with higher mental functions, such as the cortex and hippocampus [1]. The US Food and Drug Administration (FDA) approved four AChE inhibitor drugs such as tacrine, donepezil, galantamine, rivastigmine and the *N*-methyl-d-aspartate (NMDA) receptor antagonist-memantine as palliative treatment for this devastating pathology [2,3]. However, it has been established that AD is multifactorial, and the hope for novel treatments of AD could be more effective using multi-target drug approaches.

Currently, clinical treatments using AChE inhibitors lead to the temporary amelioration of cognition and memory damage based on the improvement of cholinergic neurotransmission [4]. It is known that AChE has two binding sites: the catalytic site related to acetylcholine hydrolysis, and the peripheral site for Aβ interactions [5]. Taking into account that interaction of AChE with the Aβ protein gives rise to stable complexes of AChE-Aβ, which cause an increase in the neurotoxicity of Aβ fibrils, the search of dual binding site AChE inhibitors is a potential disease-modifying strategy urgently required [6]. Several potent dual binding site AChE inhibitors act simultaneously on two aspects of AD pathology, e.g., amyloid modulation and AChE inhibition [7], and they bear different cores, such as phthalimide [1], substituted indanone or indane [1,8,9,10], indole [11], alkoxybenzene [12] or *N*-benzyl-piperidine or -piperazine rings [13,14] connected to the acridine scaffold by diverse nitrogen-containing bridges, mimicking donepezil (**1**) and tacrine (**2**) moieties. Despite the high activity displayed by these derivatives, inducing AChE inhibition in a range of 0.02 to 113 nM and some of them reducing the aggregation of Aβ up to 99% at 100 µM [11], the notable mutagenic effect of the acridine nucleus, for instance, related to the frameshift mutation (insertion or deletion of bases), should be considered to preclude the incorrect reading of DNA [15]. As an alternative scaffold, quinoline preserves some structural features of acridine and is not toxic as it is present in many marketed drugs (antimalarial, CNS, analgesic, anti-inflammatory, *etc*.) and natural products, such as Cinchona alkaloids [16].

To increase the speed of the first steps in drug discovery and development, a reliable synthetic route is required to produce structural diversity in a fast and efficient manner, since the synthesis of biologically active molecules might be time consuming and costly. The click chemistry strategy, in particular the most common cyclo-addition reaction CuAAC (Copper(I)-catalysed Azide/Alkyne Cycloaddition), firstly introduced by Sharpless (2001), is easy to perform and generate 1,4-disubstituted 1,2,3-triazoles in high yields, purity and selectivity from functionalized building blocks in a convergent synthetic approach [17,18].

**Scheme 1:**
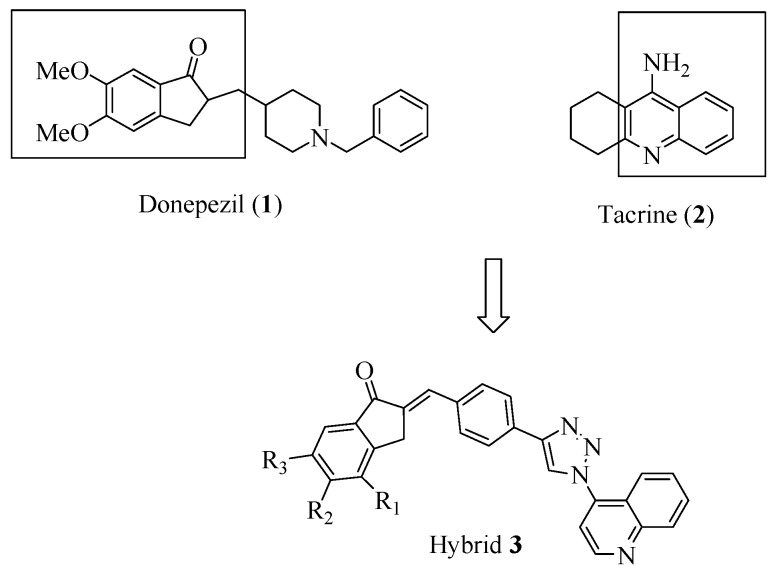
Molecular strategy related to triazole-quinoline hybrid **3**, obtained from hybridization of donepezil (**1**) and tacrine (**2**).

Based on the potent AChE inhibition displayed by some known 1,2,3-triazoles derivatives [19,20,21], we pursued the synthesis of a small library of rigid hybrid molecules (Scheme 1), mimicking tacrine (acridine) and donepezil (indanone), respectively, by a quinoline system, as a new core for recognition in the catalytic AChE site, and a dimethoxyindanone moiety, complementary at the peripheral site, connected by a sp^2^ methine-phenyltriazole bridge, employing the CuAAC reaction. The conformational constraint imposed for this linker maintains the molecules in an extended format and might avoid potential energy penalties for selective AChE interactions, in preference to BChE binding, which requires a folded conformation [22].

To assess the inhibitory activity and selectivity of the hybrids **3a**−**h**, we performed Ellman’s assays using *h*AChE and *h*BChE. In addition, to gain insight into the mechanism of action, kinetic assays and molecular modeling studies were conducted to explore the binding affinity of these compounds for both active and peripheral *h*AChE sites.

## 2. Results and Discussion

### 2.1. Chemical Synthesis

**Scheme 2:**
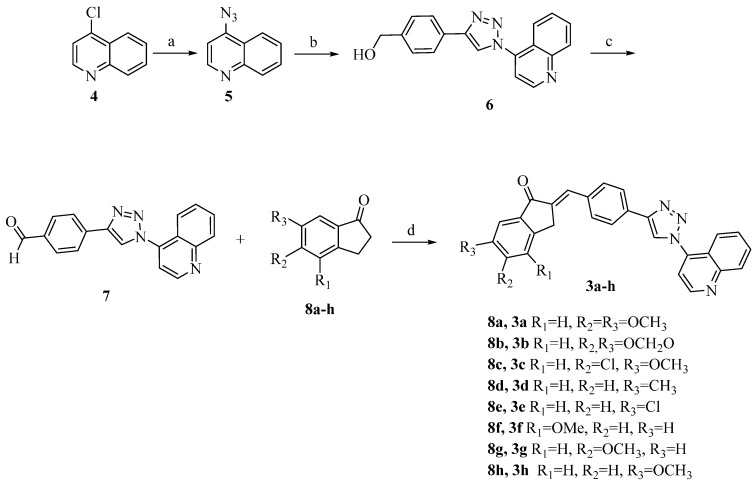
Synthesis of the hybrid compounds **3a**–**h**. Conditions: (**a**) NaN_3_, EtOH/H_2_O (1:1), reflux, 18 h, quantitative yield; (**b**) 4-ethynylbenzyl alcohol, CuSO_4_, sodium ascorbate, DMF, microwave irradiation, 70 °C, 10min, 90%; (**c**) Pyridinium chlorochromate, acetone, microwave irradiation, 60 °C, 5 min, 85%; (**d**) KOH, EtOH/DMF (10:1), room temperature, 3 h, 75%–96%.

The syntheses of hybrids **3a**–**h** were performed as shown in Scheme 2. The 4-azidoquinoline (**5**) was prepared through the replacement of quinoline chlorine **4** by the azide group (quantitative yield), and confirmed by infrared spectroscopy absorptions in 2118 cm^−1^, characteristic of the azide group. The 1,3-dipolar cycloaddition reaction, CuAAC, between **5** and commercial 4-ethynylbenzyl alcohol, afforded the corresponding 1,2,3-triazole 1,4-disubstituted alcohol with 90% yield. This reaction was performed under microwave irradiation in a short time (10 min) and the alcohol **6** was purified by column chromatographic (CC). ^1^H-NMR spectrum showed the characteristic triazole hydrogen (singlet) at 9.29 ppm, two aromatics in 7.98 and 7.47 ppm singlets corresponding to phenyl hydrogen, the quinoline hydrogens in the range 9.19–7.77 ppm, hydroxyl group in 5.31 ppm (triplet) and methylene benzylic hydrogens in 4.57 ppm (doublet).

Further oxidation reaction of this alcohol **6**, using pyridinium chlorochromate (PCC) in acetone, also under microwave irradiation for 5 min, allowed the preparation of a key aldehyde **7** in 85% yield after purification by CC. The structure was confirmed by ^1^H-NMR spectrum, which displayed the aldehyde hydrogen at 10.06 ppm (singlet), as well as the rest of the hydrogen, excepting the hydroxyl group.

The aldol condensation reactions between aldehyde **7** and the indanone enolates library (**8a**–**h**), formed in the presence of KOH in EtOH/DMF, were carried out through an E1cB elimination reaction to produce a series of conjugated triazole enone hybrids in high yield (75%–96%). In these cases, products precipitated in the reaction mixture and no further purifications were necessary. All obtained data for compound **3a**–**h** were in accordance with the expected structure, as confirmed by the ^1^H-NMR spectrum showing, in all cases, olefinic hydrogens in a range of 7.64–7.28 ppm, the methylene indanone hydrogens around 4 ppm and also all other related aromatic hydrogen. To assign the correct olefin stereochemistry, we performed the G-BIRD_R.X_-CPMG-HSQMBC [23,24] experiments to measure heteronuclear spin-spin coupling constant C-H (^3^*J*_CH_) between olefinic hydrogen and carbonyl carbon. According to Karplus equation, vicinal coupling constants present a strong correlation with the dihedral angle (θ) enabling correlation of the ^3^*J*_CH_ value with *E* (θ = 0°) or *Z* (θ = 180°) stereochemistry of the double bond [25,26]. Thus, in this case, the ^3^*J*_CH_ = 6.9 Hz was observed in the NMR experiment, which corresponds to θ around 0°, confirming the exclusive presence of isomer *E* as expected, due to the most favorable *anti* elimination in the E1cB reaction, giving rise to a more stable *E*-isomer [27].

### 2.2. Biological Evaluation

The traditional assay to screen cholinesterase inhibitors is based on the conventional UV detection of thiocholine through derivatization with Ellman’s reagent, using acetylthiocholine (ATChI) as the reactive substrate, and tacrine and donepezil as reference drugs [28]. Therefore, the preliminary assays showed the ability of some triazole-quinoline hybrids (**3a**, **3e**, **3g**, **3h**) to inhibit *h*AChE, human recombinant, in a range of 29%–55% at 100 μM. Therefore, compounds with a percentage of inhibition higher than 25% were selected for the determination of the inhibitory potency (IC_50_) and inhibition mechanism using the same enzyme. The results presented in Table 1 revealed compounds **3g** and **3h** as the most potent of the series with IC_50_ values of 114 and 109 μM, respectively. However, the activity of compound **3f** was not possible to obtain because of its similar UV absorption to 5-thio-2-nitro-benzoic acid, the product of Ellman’s enzymatic reaction, at 412 nm. Despite the IC_50_ values for hybrid triazoles being higher than the reference drugs, the inhibition values are comparable with IC_50_ described for isoquinoline analog, with 5.7% to 85.1% inhibition of *h*AChE at 100 μM [29]. We assumed that the constraint structures **3a**–**h** might not adopt the required arrangements to interact with both AChE sites as accomplished by more flexible molecules previously described, even though the linker length between the anchoring indanone and quinoline rings (8 atoms) was similar to other dual binding site inhibitors (9 to 10 atoms) [1,8,9,10,11,12,13,14] and donepezil (6 atoms). On the other hand, a remarkable selectivity was obtained for *h*AChE inhibition since none of the tested compounds showed inhibitory activity against *h*BChE. Subsequently, all active inhibitors were screened for false positive effects on BChE and AChE inhibitions and none of them fell into this category [30].

**Table 1:** Percentage of *h*AChE and *h*BChE inhibition (at 100 μM compound concentration), calculated IC_50_ values and inhibition constant (K_i_) and mechanism of the action for hybrid compounds **3a**–**e** and **3g**–**h**. Data for reference drugs donepezil (**1**) and tacrine (**2**) are also included.

Compounds	*h*AChE				*h*BChE
	% Inhibition for Assay *h*AChE (at 100 μM)	IC_50_ ± SEM (μM)	K_i_ ± SEM (μM)	Mechanism Type	% Inhibition for Assay *h*BChE (at 100 μM)
Tacrine	92.8	1.7 ± 0.1	0.07 ± 0.01	mixed-type	98.0
Donepezil	83.9	0.12± 0.02	2.2 ± 0.05	mixed-type	11.1
**3a**	29.0	128.0 ± 25	185 ± 29	ND	0
**3b**	0	ND	ND	ND	5.2
**3c**	5.40	ND	ND	ND	6.2
**3d**	9.10	ND	ND	ND	0
**3e**	37.7	146.6 ± 15	530 ± 150	ND	0
**3g**	48.1	114.0 ± 10	220 ± 60	mixed-type	0.2
**3h**	55.7	109.4 ± 14	245 ± 70	mixed-type	0

SEM: standard error of the mean; IC_50_: compound concentration required to produce the 50% of inhibition; K_i_: inhibition constant. ND: Not determined.

**Figure 1:**
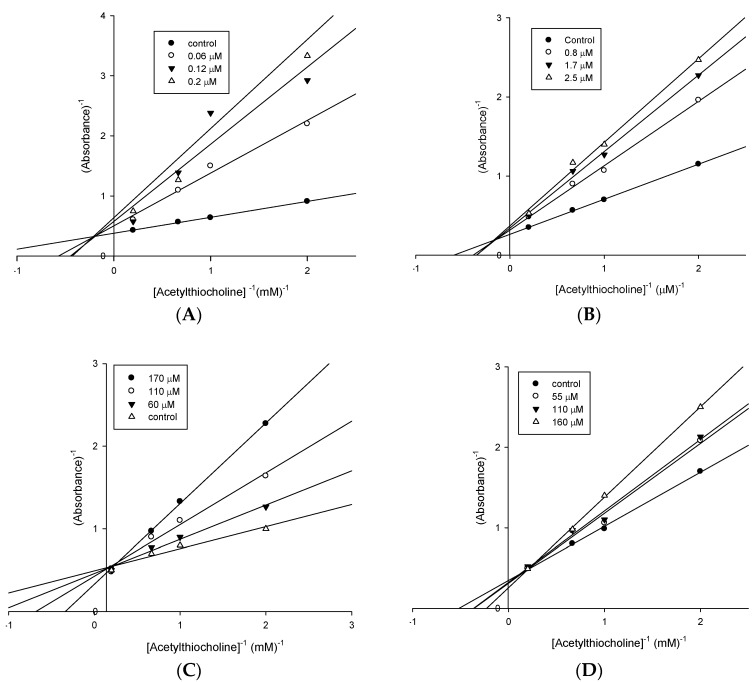
Lineweaver-Burk plots for AChE inhibiton by (**A**) donepezil, (**B**) tacrine; (**C**) compound **3g** and (**D**) compound **3h**.

The Lineweaver-Burk plots for donepezil and tacrine (Figure 1A,B) followed a mixed-type mechanism for *h*AChE, which is in accordance with published results for these well-known *h*AChE inhibitors [31,32]. Similar to standard compounds, the most active derivatives **3g**–**h** (Figure 1C,D) were tested and also followed the mixed-type mechanism as expected for the action of dual binding site inhibitors.

### 2.3. Molecular Modeling

To shed some light on how the triazole-quinoline **3h** molecule might interact with peripheral (PS) and catalytic sites (CS) of *h*AChE, and determine some potential interactions with the enzyme for further optimization, we performed docking studies using donepezil (**1**), as reference (PDB code 4EY7) [33] and GOLD software [34,35,36].

**Figure 2:**
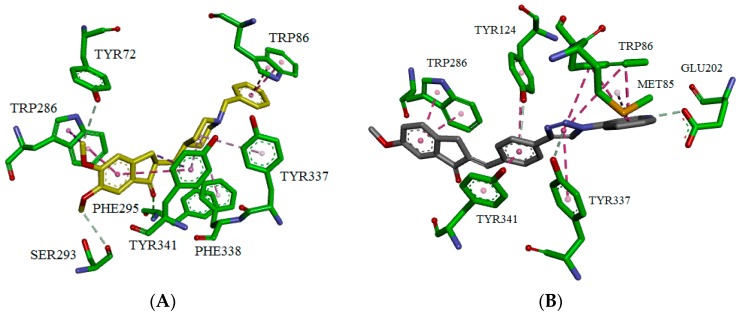
Molecular docking derived binding pose of donepezil (**A**) and compound **3h** (**B**) in the binding sites (CS and PAS) of AChE enzyme. GOLD software was used to derive the binding mode and the picture was generated from DS Visualizer software.

Despite the importance of AChE catalytic triad residues, SER203, GLU334 and HIS447, the interactions with two tryptophan residues, one located at peripheral site (TRP286) and the other in the catalytic cleft (TRP86), are crucial to achieve strong inhibitory effects by dual binding site inhibitors [7]. Therefore, docking results showed similar binding modes for donepezil (**1**) and the inhibitor **3h** to *h*AChE. This includes a π-π stacking interaction between the indanone ring of both compounds with TRP286, and a second π-π stacking interaction between the benzyl group of donepezil (**1**) or the quinoline ring of compound **3h** with TRP86, Figure 2. Additionally, although some other interactions were also observed for derivative **3h** at the middle of the gorge, with aromatic tyrosine residues, such as TYR124, TYR337, and TYR341, these are different from those present in donepezil and may be responsible for the activity variations. The binding mode here reported suggests that these triazole-quinoline compounds behave as dual binding site AChE inhibitors and show some key structural points to be considered in future optimization.

Based on the mode of action and *h*AChE selectivity, in addition to the straightforward synthetic strategy, these triazole-quinoline derivatives can be considered a new structural scaffold for *h*AChE inhibition for further chemical modifications.

## 3. Experimental Section

### 3.1. General Information

All chemicals were purchased as reagent grade and used without further purification. Solvents were dried according to standard procedures [37]. Alkynes were purchased from Sigma-Aldrich, St. Louis, MO, USA. Reactions were monitored by thin layer chromatography (TLC) on pre-coated silica gel aluminum plates (60 GF254, Merck, Kenilworth, NJ, USA) and compounds were visualized by ultraviolet light (254 nm) (Spectroline CM-10) or iodine vapor. The microwave-assisted reactions were performed in sealed tubes on a CEM Discover^®^ Microwave System (CEM Corporation, Mattheus, NC, USA). Nuclear magnetic resonance spectra were recorded on Bruker Advance DRX 300 (300 MHz), DPX 400 (400 MHz) or DPX 500 (500 MHz) spectrometers (Bruker, Billerrica, MA, USA) and chemical shifts are expressed in ppm (δ), using tetramethylsilane (TMS) or the residual non-deuterated solvent signal as an internal standard. Assignments were supported by COSY (Homonuclear Correlation Spectroscopy) HSQC (Heteronuclear Single Quantum Correlation), HMBC (Heteronuclear Multiple Bond Correlation) and the G-BIRD_R.X_-CPMG-HSQMBC spectra when necessary. Accurate mass electrospray ionization mass spectra (ESI-HRMS) were obtained on a Bruker Daltonics MicroOTOF II ESI-TOF mass spectrometer (Bruker).

### 3.2. Chemistry

#### 3.2.1. 4-Azidoquinoline (**5**)

A mixture of 4-choroquinoline (113 mg; 0.69 mmol, 1.0 equiv) and sodium azide (184 mg; 2.83 mmol, 4.0 equiv) in H_2_O (8 mL) and EtOH (8 mL) was stirred under reflux for 18 h. Subsequently, the ethanol was removed under reduced pressure. The aqueous phase was extracted with DCM (2 × 15 mL). The organic phase was dried over anhydrous MgSO_4_, filtered, and concentrated in vacuum. Compound **5** (115 mg; 0.68 mmol; 98%) was obtained as a yellow solid that required no further purification. IR (neat): 2118 cm^−1^ (N_3_). ^1^H-NMR (400 MHz, CDCl_3_): δ_H_ ppm 8.77 (1H, d, *J* = 4.9 Hz, H3); 8.01 (2H, dd, *J* = 7.9; 1.1 Hz, H5 and H8); 7.69 (1H, ddd, *J* = 8.4; 6.9; 1.2 Hz, H6); 7.49 (1H, ddd, *J* = 8.4; 6.9; 1.2 Hz, H7); 7.10 (1H, d, *J* = 4.9 Hz, H2). ^13^C-NMR (400 MHz, CDCl_3_): δ_C_ ppm 150.1 (C3); 149.0 (C4); 146.4 (C1); 130.6 (C6); 129.1 (C5); 126.7 (C7); 122.3 (C8); 121.6 (C9); 108.6 (C2).

#### 3.2.2. 4-(4-(4-Hydroxymethyl-phenyl)-1*H*-1,2,3-triazol-1-yl)-quinoline (**6**)

To a solution of 4-azidoquinoline (**5**) (32.5 mg; 0,176 mmol, 1.0 equiv) and 4-ethynylbenzyl alcohol (25.6 mg, 0.194 mmol, 1.1 equiv) in DMF (0.3 mL) sodium ascorbate (3.5 mg; 0.0176 mmol, 0.1 equiv) and CuSO_4_ (0.8 mg; 0.005 mmol 0.03 equiv) were added. The reaction mixture was stirred for 10 min at 70 °C (sealed microwave tube) under microwave irradiation (100 W) and after completion toluene (20 mL) was added and the solvents were removed under reduced pressure. The crude was partitioned between EtOAc (20 mL) and H_2_O (20 mL) and the aqueous phase was extracted with EtOAc (2 × 20 mL). The organic phase was dried over anhydrous MgSO_4_, filtered, and concentrated in vacuum. The crude mixture was purified by column chromatography on silica gel in a EtOAc/hexane (7:3) mixture as eluent, to support the compound **6** (48 mg; 158 mmol; 90%). ^1^H-NMR (400 MHz, CDCl_3_): δ_H_ ppm 9.29 (1H, s, H10); 9.16 (1H, d, *J* = 4.6 Hz, H3); 8.24 (1H, d, *J* = 8.4 Hz, H5); 8.07 (1H, d, *J* = 8.4 Hz, H8); 7.98 (2H, d, *J* = 8.1 Hz, H13); 7.97–7.91 (1H, m, H6); 7.90 (1H, d, *J* = 4.6 Hz, H2); 7.77 (1H, t, *J* = 8.2 Hz, H7); 7.47 (2H, d, *J* = 8.1 Hz, H14); 5.31 (1H, t, *J* = 5.7 Hz, OH); 4.57 (2H, d, *J* = 5.7 Hz, H16). ^13^C-NMR (400 MHz, CDCl_3_): δ_C_ ppm 150.9 (C3); 149.1 (C4); 147.1 (C11); 142.9 (C1); 140.3 (C15); 130.7 (C6); 129.5 (C5); 128.5 (C7); 128.3 (C12); 127.0 (C14); 125.3 (C13); 123.4 (C10); 123.2 (C8); 121.6 (C9); 116.7 (C2); 62.6 (C16). ESI-MS *m*/*z*: Calcd for C_18_H_14_N_4_O: 302.1168 [M]^+^; found *m*/*z* 303.1235 [M + H]^+^; 325.1053 [M + Na]^+^.

#### 3.2.3. 4-(1-(Quinolin-4-yl)-1*H*-1,2,3-triazol-1-yl)-benzaldehyde (**7**)

To a solution of compound **6** (32.7 mg; 0.108 mmol, 1 equiv) in 2 mL of acetone, PCC (35.3 mg; 0.164 mmol; 1.5 equiv) was added. The reaction mixture was stirred for 5 min at 60 °C (sealed microwave tube) under microwave irradiation (200 W) and, after completion of the reaction (monitored by TLC), it was quenched with 0.5 mL of methanol before the solvents were removed under reduced pressure. The crude mixture was purified by column chromatography on silica gel in EtOAc as eluent, to support the compound **7** (27.5 mg; 0,092 mmol; 85%). ^1^H-NMR (400 MHz, CDCl_3_): δ_H_ ppm 10.06 (1H, s, H16); 9.51 (1H, s, H10); 9.17 (1H, d, *J* = 4.6 Hz, H3); 8.25 (3H, d, *J* = 8.4 Hz, H5 and H14); 8.08 (2H, d, *J* = 8.5 Hz, H13); 8.09–8.04 (1H, m, H8); 7.98 (1H, ddd, *J* = 8.5; 6.9; 1.3 Hz, H6); 7.93 (1H, d, *J* = 4.6 Hz, H2) 7.78 (1H, ddd, *J* = 8.5; 6.9; 1.2 Hz, H7). ^13^C-NMR (400 MHz, CDCl_3_): δ_C_ ppm 192.6 (C16); 151.0 (C3); 149.1 (C4); 146.0 (C11); 140.2 (C1); 135.8 (C15); 135.6 (C6); 130.8 (C5); 130.4 (C14); 129.6 (C7); 128.6(C6); 126.0 (C13); 125.4 (C10); 123.1 (C8); 121.6 (C9); 117.0 (C2).

#### 3.2.4. General Procedure for the Preparation of **3a**–**h**

To a solution of compound **7** (0.040 mmol, 1.0 equiv) and commercial indanone (**8a**–**h**) (0.044 mmol, 1.1 equiv) in EtOH (1 mL) and DMF (0.5 mL), 150 μL of ethanolic solution 4% KOH was added.Afterwards, the mixture was stirred at room temperature for 3 h. The produced solid was filtered and washed with water and acetone. Compounds **3a**–**h** were obtained as solids that required no further purification.

*(E)-5,6-Dimethoxy-2-(4-(1-(quinolin-4-yl)-1H-1,2,3-triazol-4-yl)benzylidene)-2,3-dihydro-1H-inden-1-one* (**3a**). ^1^H-NMR (400 MHz, DMSO): δ_H_ ppm 9.44 (1H, s, H10); 9.18 (1H, d, *J* = 4.6, H3); 8.25 (1H, d, *J* = 8.5 Hz, H5); 8.14 (1H, d, *J* = 8.3, H14); 8.06 (1H, d, *J* = 8.3, H8); 7.98–7.92 (3H, m, H2, H6 and H13); 7.79 (1H, t, *J* = 7.3; H7); 7.50 (1H, s, H16); 7.25 (2H, s, H22 and H23); 4.09 (1H, s, H21); 3.92 (1H, s, H27); 3.85 (1H, s, H26). ^13^C-NMR (400 MHz, CDCl_3_): δ_C_ ppm 191.8 (C18); 155.4 (C24); 151.0 (C3); 149.4 (C25); 149.1(C4); 146.5 (C12); 145.2 (C19); 140.3 (C20); 136.3 (C15); 135.2 (C1); 131.3 (C13); 130.8 (C6); 130.7 (C17); 130.5 (C16); 130.0 (C9); 129.6 (C5); 128.6 (C7); 125.9 (C14); 125.0 (C10); 124.5 (C3); 123.1 (C8); 121.6 (C11); 116.9 (C2); 108.1 (C23); 104.6 (C22); 56.0 (C27); 55.7 (C26); 31.7 (C21). ESI-MS *m*/*z* calcd for C_29_H_22_N_4_O_3_: 474.1692, found *m*/*z* 475.1759 [M + H]^+^; *m*/*z* 497.1579 [M + Na]^+^.

*(E)-5,6-Methilenedioxy-2-(4-(1-(quinolin-4-yl)-1H-1,2,3-triazol-4-yl)benzylidene)-2,3-dihydro-1H-inden-1-one* (**3b**). ^1^H-NMR (300 MHz, DMSO) δ_H_ ppm 9.44 (1H, s, H10), 9.18 (1H, d, *J* = 4.9 Hz, H3), 8.25 (1H, d, *J* = 8.3 Hz, H5), 8.14 (2H, d, *J* = 8.2 Hz, H14), 8.06 (1H, d, *J* = 7.9 Hz, H8), 7.97–7.90 (4H, m, H2, H6, H13), 7.79 (1H, t, *J* = 7.3 Hz, H7), 7.49 (1H, s, H16), 7.21 (2H, s, H22 and H23), 6.21 (2H, s, H26), 4.09 (2H, s, H18).

*(E)-5-Chloro-6-methoxy-2-(4-(1-(quinolin-4-yl)-1H-1,2,3-triazol-4-yl)benzylidene)-2,3-dihydro-1H-inden-1-one* (**3c**). ^1^H-NMR (300 MHz, DMSO) δ_H_ ppm 9.38 (1H, s, H10), 9.10 (1H, d, *J* = 4.6 Hz, H3), 8.17 (1H, d, *J* = 8.3 Hz, H5), 8.07 (2H, d, *J* = 8.2 Hz, H14), 7.98 (1H, d, *J* = 8.3 Hz, H8), 7.91–7.84 (4H, m, H2, H7 and H13), 7.74–7.68 (2H, m, H6 and H22), 7.54 (1H, s, H16), 7.37 (1H, s, H23), 4.06 (2H, s, H18), 3.89 (3H, s, H26). ESI-MS (**3c**): *m*/*z* calcd for C_28_H_19_ClN_4_O_2_: 478.1197, found *m*/*z* 517.0825 [M + K]^+^.

*(E)-6-Methyl-2-(4-(1-(quinolin-4-yl)-1H-1,2,3-triazol-4-yl)benzylidene)-2,3-dihydro-1H-inden-1-one* (**3d**). ^1^H-NMR (300 MHz, DMSO) δ_H_ ppm 9.45 (H10, s, 1H,), 9.18 (1H, d, *J* = 4.6 Hz, H3), 8.25 (1H, d, *J* = 8.9 Hz, H5), 8.14 (2H, d, *J* = 8.3 Hz, H14), 8.06 (1H, d, *J* = 8.3 Hz, H8), 7.97–7.92 (4H, m, H2, H6 and H13), 7.78 (1H, t, *J* = 7.6 Hz, H7), 7.62–7.54 (4H, m, H16, H22, H23 and H24), 4.16 (2H, s, H18), 2.41 (3H, s, H26). ESI-MS (**3d**): *m*/*z* calcd for C_28_H_20_N_4_O_2_: 428.1637, found *m*/*z* 429.2378 [M + H]^+^; *m*/*z* 467.1268 [M + K]^+^.

*(E)-6-Chloro-2-(4-(1-(quinolin-4-yl)-1H-1,2,3-triazol-4-yl)benzylidene)-2,3-dihydro-1H-inden-1-one* (**3e**). ^1^H-NMR (300 MHz, DMSO) δ_H_ ppm 9.47 (1H, s, H10), 9.18 (1H, d, *J* = 4.6 Hz, H3), 8.26 (1H, d, *J* = 8.6 Hz, H5), 8.16 (2H, d, *J* = 8.4 Hz, H14), 8.06 (H, d, *J* = 8.3 Hz, H8), 7.98 (2H, d, *J* = 8.6 Hz, H6 and H7), 7.94 (1H, d, *J* = 4.6 Hz, H2), 7.81–7.77 (3H, m, H22, H23 and H24), 7.65 (1H, s, H16), 4.22 (1H, s, H18).

*(E)-4-Methoxy-2-(4-(1-(quinolin-4-yl)-1H-1,2,3-triazol-4-yl)benzylidene)-2,3-dihydro-1H-inden-1-one* (**3f**). ^1^H-NMR (500 MHz, DMSO) δ_H_ ppm 9.42 (1H, s, H10), 9.16 (1H, d, *J* = 4.6 Hz, H3), 8.24 (1H, d, *J* = 8.4 Hz, H5), 8.14 (2H, d, *J* = 8.1 Hz, H14), 8.04 (1H, d, *J* = 8.3 Hz, H8), 7.97–7.91 (4H, m, H2, H6 and H13), 7.61 (1H, t, H7), 7.59 (1H, s, H16), 7.48 (1H, t, H25), 7.38 (1H, d, *J* = 7.7 Hz, H23), 7.31 (1H, d, *J* = 7.7 Hz, H24), 4.03 (2H, s, H18), 3.94 (3H, s, H26). ESI-MS (**3f**): *m*/*z* calcd for C_28_H_20_N_4_O_2_: 444.1586, found *m*/*z* 483.1152 [M + K]^+^.

*(E)-5-Methoxy-2-(4-(1-(quinolin-4-yl)-1H-1,2,3-triazol-4-yl)benzylidene)-2,3-dihydro-1H-inden-1-one* (**3g**). ^1^H-NMR (300 MHz, DMSO) δ_H_ ppm 9.45 (1H, s, H10), 9.18 (1H, d, *J* = 4.6 Hz, H3), 8.26 (1H, d, *J* = 8.4 Hz, H5), 8.15 (2H, d, *J* = 8.3 Hz, H14), 8.07 (1H, d, *J* = 7.9 Hz, H8), 7.99–7,93 (3H, m, H2, H6 and H13), 7.80 (1H, t, *J* = 7.7 Hz, H7), 7.76 (1H, d, *J* = 8.4 Hz, H23), 7.53 (1H, s, H16), 7.24 (1H, d, *J* = 2.0 Hz, H22), 7.07 (1H, dd, *J* = 8.5; 2.0 Hz, H25), 4.17 (2H, s, H18), 3.92 (3H, s, H26). ESI-MS (**3g**): *m*/*z* calcd for C_28_H_20_N_4_O_2_: 444.1586, found *m*/*z* 483.1219 [M + K]^+^.

*(E)-6-Methoxy-2-(4-(1-(quinolin-4-yl)-1H-1,2,3-triazol-4-yl)benzylidene)-2,3-dihydro-1H-inden-1-one* (**3h**). ^1^H NMR (500 MHz, DMSO) δ_H_ ppm 9.35 (1H, s, H10), 9.16 (1H, d, *J* = 4.5 Hz, H3), 8.25 (1H, d, *J* = 8.7 Hz, H5), 8.15 (2H, d, *J* = 8.2 Hz, H14), 8.05 (1H, d, *J* = 8.5 Hz, H8), 7.96–7,93 (3H, m, H6 and H13), 7.89 (1H, d, *J* = 4.5 Hz, H2), 7.78 (1H, t, *J* = 7.7 Hz, H7), 7.62–7.60 (2H, m, H16 and H22), 7.33 (1H, dd, *J* = 8.3; 2.5 Hz, H24), 7.28 (1H, s, H23), 4.12 (2H, s, H18), 3.86 (3H, s, H26). ESI-MS HRMS (ESI) (**3h**): *m*/*z* calcd for C_28_H_20_N_4_O_2_: 444.1586, found *m*/*z* 483.1235 [M + K]^+^.

### 3.3. Biological Assays

The enzymes acetylcholinesterase (*h*AChE EC 3.1.1.7, 1000 units mg^−1^ from human recombinant, expressed in HEK 293 cells) and butyrylcholinesterase (*h*BChE EC 3.1.1.8, 50 units mg^−1^ from human erythrocytes) as lyophilized powder; their substrates acetylthiocholine iodide (ACThI) and butyrylthiocholine iodide (BTChI); 5,5′-dithiobis(2-nitrobenzoic acid) (Ellman’s reagent or DTNB); donepezil hydrochloride monohydrate; tacrine (9-amino-1,2,3,4-tetrahydroacridine hydrochloride hydrate) were all obtained from Sigma-Aldrich (St. Louis, MO, USA). Buffer components and all of the chemicals used were all analytical grade materials, and were obtained from Sigma, Merck (Darmstadt, Germany), Synth (São Paulo, Brazil) or Acros (Geel, Belgium). The water used in all of the preparations was purified with a Millipore Milli-Q^®^ system (Millipore, São Paulo, Brazil). Stock solutions (1 mM) of the evaluated inhibitors were prepared and diluted with dimethylsulfoxide (DMSO)/water (50% *v*/*v*) to give the desired concentration ranges.

The ligand assays were carried out in accordance with the literature [28,29], albeit with some modifications. Briefly, the assays were carried out using a 96-well microplate with an Elisa microplate reader. To a final volume of 250 µL, each well was filled with: 125 µL of Ellman’s reagent (3 mM in 0.1 mM phosphate buffer pH 7.4); 50 µL of buffer TRIS (50 mM, pH 8.0); 25 µL of enzyme solution at the final concentration 0.28 U/mL (in 0.1 mM phosphate buffer pH 7.4); 25 µL of the ACThI (15 mM) for AChE and 25 µL of the BCThI (15 mM) for BChE; and 25 µL of each inhibitor sample (1 mM), final concentration at 100 µM. The microplate was shaken for 10 s followed by reading the absorbance at 412 nm at 30 s intervals for two minutes. The negative control (compound and absence of ACThI) and a positive control (ACThI and absence of compound) were carried out.

The inhibition percentage was obtained by comparing the absorbance in the presence of inhibitor (A_i_) and in the absence of inhibitor (A_0_) according to the expression, % Inhibition = 100 − [(A_i_/A_0_) × 100]. The assays were carried out in duplicate. Tacrine and donepezil were used as standard inhibitors *h*AChE.

For the inhibitory potency (IC_50_) assay, increasing concentrations of the 25 µL of each inhibitor sample (0.05–50 µM) for standard inhibitors and (25–500 µM) for the ligands were prepared from a 1 mM stock solution. IC_50_ values were independently determined by performing rate measurements for at least six concentrations of the target inhibitor. The nonlinear regression parameters were calculated, and the IC_50_ was extrapolated.

For the steady-state inhibition constant (K_i_) and mechanism of the action assay, ranging concentrations of the 25 µL of ATChI solutions (0.5, 5.0, 10, 15 and 50 mM) containing fixed ligands concentrations (from 0.06 to 2.5 µM for standard inhibitors and 55 to 250 µM for the ligands) were carried out. Reciprocal plots of 1/absorbance *versus* 1/[ACThI] were constructed and the constant K_i_ can be determined from the replots of primary reciprocal plot data.

#### False-Positive Effects on BChE and AChE Inhibition in the Thin Layer Chromatography (TLC) Assay Based on Ellman’s Method

Each compound sample (2.5 μL) was eluted on a chromatographic silica gel 60 plate using CHCl_3_:MeOH:H_2_O 65:30:5 (*v*:*v*) as the mobile phase. After drying, the plates were sprayed with a solution containing AChE (0.704 mg), BSA (0.025 g), and ACThI (0.00723 g) in Tris (19 mol·L^−1^, pH 8, adjusted with HCl 10% *v*:*v*), previously incubated at 37 °C for 15 min, followed by addition of Ellman’s reagent prepared in Tris (19 mol·L^−1^, pH 8, adjusted with HCl 10% *v*:*v*) for the assays with AChE. As for the assays with BChE, BChE itself and its substrate BCThI were used at the same concentrations listed above. This assay was carried out as described in the literature [30].

### 3.4. Docking Procedures

Molecular docking studies were performed using GOLD 5.2 [34,35] with *h*AChE complexed with donepezil (PDB code 4EY7). Top-ranked orientations were selected by GOLD via the ChemPLP empirical energy function [36]. In each calculation, 10 orientations (docking runs) were obtained, and the highest score (top-ranked) was selected for each compound. The simulations were performed inside a sphere of 10 Å radius in a point centered under *N*-piperidine atom of donepezil.

## 4. Conclusions

The multi-target treatment for AD is one of the most promising strategies to combat this complex and devastating neurodegenerative disorder urgently in need of an effective treatment. Among them, dual binding site AChE inhibitors showed the advantage of targeting a druggable pharmacological enzyme, modulating both symptomatic and disease-modifying symptoms as potent β-amyloid modulators [38]. Herein, we have designed and synthesized a novel chemical class of triazole-quinoline derivatives, able to fit in peripheral and catalytic sites of AChE, thus providing a new chemical scaffold of selective dual binding site AChE inhibitors. Moreover, these compounds can be easily prepared with the click chemistry approach. Biological *in vitro* studies confirm the therapeutic value of these compounds. Further developments are in progress to optimize these new triazole-quinoline derivatives as potential disease-modifying drug candidates for Alzheimer’s disease treatment in the future.
